# Ultrasound-Detected Salivary Gland and Joint Inflammation Strongly Reflect Patient-Perceived Symptom Burden in Primary Sjögren’s Syndrome: A Cross-Sectional Multicenter Study

**DOI:** 10.3390/biomedicines14040819

**Published:** 2026-04-03

**Authors:** Tanya Sapundzhieva, Lyubomir Sapundzhiev, Plamen Todorov, Martin Mitev, Anastas Batalov

**Affiliations:** 1 Department of Internal Diseases, Medical Faculty, Medical University of Plovdiv, 4002 Plovdiv, Bulgaria; drtodorovplamen@gmail.com (P.T.); m.mitev97@gmail.com (M.M.); abatalov@hotmail.com (A.B.); 2 Rheumatology Department, University Hospital ‘Pulmed’, 4002 Plovdiv, Bulgaria; sapoundjiev@abv.bg; 3 Rheumatology Clinic, University Hospital ‘Sofiamed’, 1113 Sofia, Bulgaria; 4 Rheumatology Clinic, University Hospital ‘Kaspela’, 4000 Plovdiv, Bulgaria

**Keywords:** Sjogren syndrome, ultrasonography, salivary glands, synovitis

## Abstract

**Aims.** To investigate the relationship between ultrasound (US)-detected parenchymal abnormalities in the major salivary glands (MSG), joint and tendon inflammation, and systemic disease activity in patients with primary Sjögren’s syndrome (pSS). **Patients and methods.** This cross-sectional, multicenter study enrolled 60 patients with pSS and 20 healthy controls (HCs). Systemic disease activity was evaluated using the EULAR Sjögren’s Syndrome Disease Activity Index (ESSDAI), while symptom burden was assessed with the EULAR Sjögren’s Syndrome Patient Reported Index (ESSPRI). MSG evaluation included bilateral gray-scale (GS) and power Doppler (PDUS) assessment of the parotid and submandibular glands using a semi-quantitative 0–3 scoring system. Musculoskeletal ultrasound (MSUS) assessment comprised bilateral examination of the wrists, second to fifth metacarpophalangeal (MCP) and proximal interphalangeal (PIP) joints, the fourth extensor wrist compartment, and the flexor tendons of the second to fifth fingers for GS and PD-detected synovitis and tenosynovitis, also scored semi-quantitatively. Recorded outcomes included GS and PD synovitis scores, total synovitis score, tenosynovitis score, GS and PD glandular scores, and total glandular score. **Results.** Synovitis was most frequently detected in the wrists, followed by the second PIP joint. Subclinical synovitis—defined as a GSUS synovitis score > 0 in a joint without clinical swelling—was detected in 66.7% (n = 28) of patients with pSS. No significant correlations were found between joint US scores and salivary gland US scores. ESSPRI showed moderate positive correlations with both the GS synovitis score (*p* = 0.002) and the total synovitis score (*p* = 0.003), as well as significant positive correlations with all salivary gland US scores: GS (*p* < 0.001), PD (*p* = 0.002), and total glandular score (*p* < 0.001). ESSDAI demonstrated only a weak positive correlation with the GS salivary gland score (*p* = 0.030). **Conclusions.** In patients with pSS, the extent of US-detected MSG parenchymal abnormalities does not reflect systemic disease activity and does not correlate with US-detected joint synovitis. In contrast, patient-reported symptom burden is associated with both joint inflammation and MSG parenchymal changes on US. Larger studies are needed to further define the role of salivary gland and joint US in evaluating disease activity in pSS.

## 1. Introduction

Sjogren syndrome (SS) is an autoimmune disease with a highly variable clinical presentation, ranging from localized glandular involvement to widespread multi-organ disease. It may occur as an independent nosological entity (primary SS, pSS) or in association with other rheumatic disorders, most commonly rheumatoid arthritis, systemic lupus erythematosus, and systemic sclerosis [1]. The estimated prevalence ranges from 0.01% to 0.05% of the general population [1]. Up to 40% of patients with pSS develop systemic manifestations, including musculoskeletal, neurological, dermatological, and respiratory involvement [2].

Since its validation in 2009, the European League Against Rheumatism (EULAR) Sjögren’s Syndrome Disease Activity Index (ESSDAI) has become the most widely used tool for assessing systemic disease activity in both routine rheumatology practice and clinical trials [3]. ESSDAI is sensitive to change and useful for monitoring treatment response, although it has two notable limitations: it does not incorporate glandular function or patient-reported outcomes [4]. Complementing this, the EULAR Sjögren’s Syndrome Patient Reported Index (ESSPRI) provides a simple assessment of the major symptoms experienced by SS patients—fatigue, dryness, and pain—using a 0–10 visual analogue scale (VAS) [5]. While ESSDAI offers an objective evaluation of systemic activity, ESSPRI captures the patient’s subjective symptom burden, making the combined use of both indices essential for a comprehensive assessment of disease status.

Ultrasound (US) has gained increasing prominence in rheumatology over the past three decades due to its accessibility, cost-effectiveness, absence of ionizing radiation, and ability to provide rapid, multiplanar, multi-site, and dynamic evaluation [6,7]. Initially used primarily for musculoskeletal assessment, its applications have expanded to include imaging of muscles, salivary glands, peripheral nerves, lungs, vessels, and skin [6,7,8].

In pSS, US can be employed to evaluate the major salivary glands (MSG)—the parotid (PG) and submandibular glands (SMG)—as well as joint and tendon inflammation and, in some cases, lung involvement such as interstitial lung disease [8]. Salivary gland US (SGUS) has demonstrated high sensitivity and specificity for diagnosing pSS, correlates well with labial salivary gland biopsy findings, aids in early detection of lymphoma, guides core needle biopsy, and is responsive to systemic treatment [9,10,11,12,13]. Recently, the Outcome Measures in Rheumatology Clinical Trials (OMERACT) group developed a semi-quantitative scoring system for gray-scale (GS) and power Doppler (PD) US, grading abnormalities from 0 to 3 based on parenchymal inhomogeneity and the presence of hypo- or anechoic areas, with grade ≥ 2 considered pathological [14,15].

The aim of the present study was to examine the relationship between US findings in the PG and SMG, musculoskeletal (MS) US findings (synovitis and tenosynovitis), and systemic disease activity in patients with pSS. To achieve this, we performed correlation analyses between salivary gland US scores, joint and tendon US scores, and systemic disease activity indices, specifically ESSDAI and ESSPRI.

## 2. Patients and Methods

### 2.1. Patients

This cross-sectional, multicenter study was conducted at the University Hospital “Kaspela” and the University Hospital “Pulmed” and included 60 consecutive patients with primary Sjögren’s syndrome (pSS), classified according to the 2016 American College of Rheumatology (ACR)/European League Against Rheumatism (EULAR) criteria, along with 20 healthy control subjects (HCs) [16]. The recruitment period extended from January 2024 to September 2025.

The study protocol was approved by the Local Ethics Committee of the University Hospital “Pulmed” (Date: 20 December 2023; Protocol №3). All participants received verbal and written information about the study and provided written informed consent in accordance with the World Medical Association Declaration of Helsinki (revised 2000, Edinburgh) prior to enrollment.

Inclusion criteria for patients were: (1) diagnosis of pSS according to the 2016 ACR/EULAR criteria [16]; (2) presence of systemic symptoms beyond sicca symptoms, fatigue, and pain; (3) stable treatment for at least three months prior to inclusion; and (4) ability to provide written informed consent.

The presence of systemic symptoms beyond sicca symptoms, fatigue, and pain was required to ensure adequate variability in systemic disease activity within the cohort. This approach was necessary to enable meaningful correlation analyses with ESSDAI and to avoid a sample composed predominantly of patients with minimal or purely glandular disease activity.

Exclusion criteria were as follows: (1) Presence of any other rheumatic disease (i.e., secondary SS); (2) Any change in treatment within the preceding three months; (3) Corticosteroid injections administered to the joints or tendons of the hands within the previous three months, and (4) Presence of severe concomitant illnesses.

HCs were required to have: (1) no sicca symptoms affecting the eyes or mouth; (2) no prior or current diagnosis of a rheumatic disease; (3) no history of pain in the small joints of the hands; (4) no clinical evidence of joint deformities or swelling in the hands; (5) no history of infection or trauma involving the hand joints; and (6) no conditions that could mimic or interfere with imaging findings, such as prior head/neck radiation therapy, hepatitis C virus infection, acquired immunodeficiency syndrome (AIDS), sarcoidosis, amyloidosis, IgG4-related disease, or graft-versus-host disease.

### 2.2. Methods

#### 2.2.1. Physical Examination

Each patient underwent a comprehensive clinical examination performed by a single physician (MM), with documentation of the status praesens. The assessment included a general physical evaluation and examination of the head and neck region, lymph nodes, and the pulmonary, cardiovascular, gastrointestinal, and genitourinary systems. Joint evaluation was conducted by the same physician (MM), who assessed for swelling, tenderness, and deformities.

The intensity of joint pain, patient global assessment of disease activity (PtGA), and the severity of dryness and fatigue were recorded using a 0–10 cm visual analogue scale (VAS). Additional clinical data included symptom duration and morning stiffness, a history of minor salivary gland biopsy, and details of previous and current treatments.

Systemic disease activity was assessed using the ESSDAI, which comprises twelve weighted domains. An ESSDAI score < 5 indicates low systemic activity, scores between 5 and 13 reflect moderate activity, and scores ≥ 14 denote high activity [3]. Patient-reported outcomes were evaluated using the ESSPRI, which measures fatigue, pain, and dryness on a 0–10 cm VAS [5].

#### 2.2.2. Paraclinical Tests

Laboratory tests. All patients underwent blood sampling for comprehensive laboratory evaluation. The following parameters were assessed: Hematologic tests: full and differential blood count; Renal function: serum creatinine (44–135 µmol/L), urea (1.7–8.3 mmol/L), uric acid (148–428 µmol/L), creatinine clearance (mL/min), urine sediment, and 24 h proteinuria (<150 mg); Liver function: aspartate aminotransferase (AST, 0–35 U/L), alanine aminotransferase (ALT, 0–45 U/L), and gamma-glutamyl transferase (GGT, 5–50 U/L); Acute-phase reactants: erythrocyte sedimentation rate (ESR) and C-reactive protein (CRP; reference range 0–5.0 mg/L). Immunologic assessment included: anti-nuclear antibodies (ANA) by indirect immunofluorescence (IIF), considered negative at titers < 1:80; anti-SS-A (Ro52) antibodies (0–10); anti-SS-B (La) antibodies (0–10); IgM rheumatoid factor (IgM-RF; 0–20 U/L); anti-citrullinated protein antibodies (ACPA; 0–20 U/L); complement fractions C3 (0.75–1.65 g/L) and C4 (0.20–0.65 g/L) and total serum IgG (6.0–16.0 g/L).

#### 2.2.3. US Examination

The US examinations were performed by two EULAR-certified US experts (TS, PT), following the 2017 EULAR recommendations for musculoskeletal ultrasound and the 2019 OMERACT guidelines for salivary gland ultrasound [14,17]. Both examiners were blinded to all clinical assessment findings and laboratory results. To assess the consistency of US scoring, a reliability analysis was performed on a subset of examinations. TS and PT independently evaluated stored images of the parotid and submandibular glands, blinded to clinical data and to each other’s readings.

Prior to the study, both sonographers participated in a calibration session in which they jointly reviewed and scored 10 representative cases covering the full range of GS and PD findings. Discrepancies were discussed until a consensus on scoring rules was reached, and these rules were then applied in all subsequent independent readings.

For inter-reader reliability, the scores assigned by the two sonographers were compared for the same set of 20 examinations. For intra-reader reliability, each sonographer re-evaluated the same examinations after a 2-week washout period, in random order, and remained blinded to their initial scores. Inter- and intra-reader agreement were quantified using weighted Cohen’s kappa with quadratic weights. Kappa values were interpreted using conventional thresholds (e.g., <0.40 poor, 0.41–0.60 moderate, 0.61–0.80 substantial, >0.80 almost perfect).

#### 2.2.4. Salivary Gland Ultrasound (SGUS)

**Figure 1:**
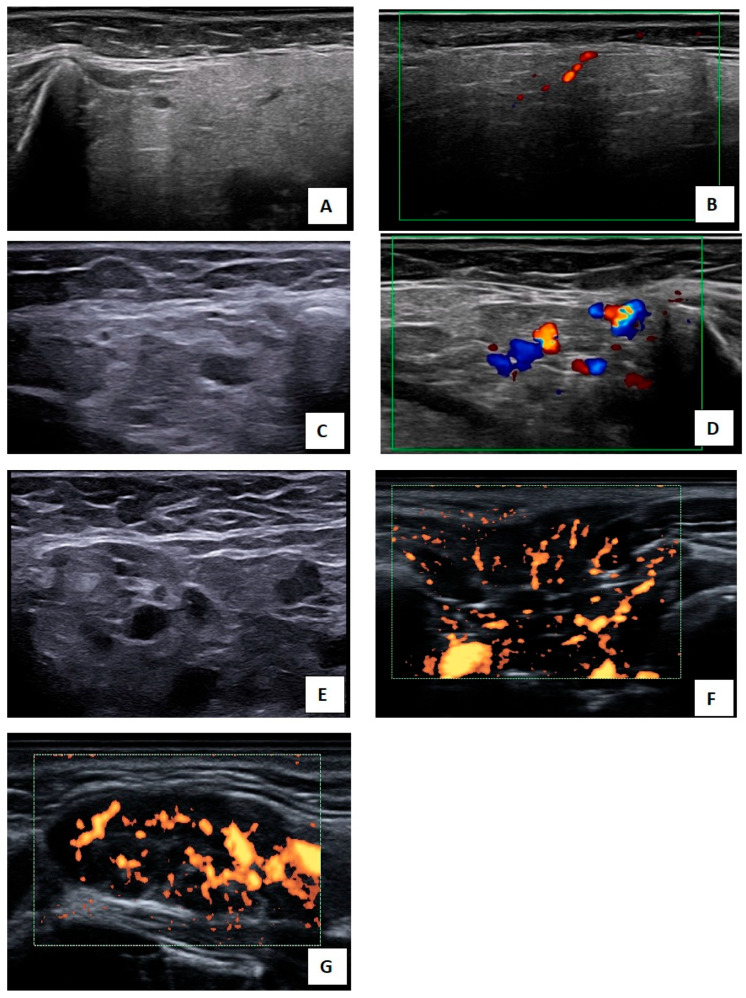
Semi-quantitative scoring of salivary gland abnormalities on GSUS (OMERACT scoring) and PDUS (Hočevar scoring). (**A**)—Grade 1 changes in the parotid gland on GSUS; (**C**)—Grade 2 changes in the parotid gland on GSUS; (**E**)—Grade 3 changes in the parotid gland on GSUS; (**B**)—Grade 1 changes in the parotid gland on PDUS; (**D**)—Grade 2 changes in the parotid gland on CDUS; (**F**)—Grade 3 changes in the parotid gland on PDUS; (**G**)—Grade 3 changes in the submandibular gland on PDUS.

The patient was examined in a supine position with the neck extended. The parotid glands were assessed in both transverse and longitudinal planes, while the submandibular glands were evaluated only in the longitudinal plane. Salivary gland abnormalities were scored according to the four-grade OMERACT system, where grade 0 represents normal parenchyma, grade 1 indicates mild inhomogeneity without anechoic or hypoechoic areas, grade 2 reflects moderate inhomogeneity with focal anechoic or hypoechoic areas, and grade 3 denotes diffuse inhomogeneity with multiple anechoic or hypoechoic areas throughout the gland or a predominantly fibrotic appearance [14]—Figure 1. PD activity was graded semi-quantitatively from 0 to 3, with grade 0 indicating absence of Doppler signal, grade 1 focal signals, grade 2 diffuse signals involving less than half of the glandular parenchyma, and grade 3 diffuse signals involving more than half [18]. The salivary gland US examination, including documentation, required approximately fifteen minutes.

MSUS was then performed bilaterally on the radiocarpal and intercarpal joints, the second to fifth metacarpophalangeal (MCP) and proximal interphalangeal (PIP) joints, the extensor digitorum communis tendon in the fourth extensor compartment, and the flexor tendons of the second to fifth digits. All structures were scanned in both longitudinal and transverse planes using a MyLab 7 (Esaote) device, Esaote S.p.A., Genova, Italy equipped with a high-frequency linear transducer (6–18 MHz). GS frequency was set at 18 MHz, with gain adjusted individually to optimize image quality. Meanwhile, PD settings included a 9.1 MHz frequency, a low wall filter, and a pulse-repetition frequency of 750 Hz. Joint synovitis and tenosynovitis were recorded in accordance with the 2019 OMERACT definitions [19,20,21]. Synovitis on both GS and PDUS was graded semi-quantitatively from 0 to 3 [19,20,21]. The wrist, MCP, and PIP joints were examined dorsally and palmarly, tenosynovitis of the fourth extensor compartment was assessed dorsally, and flexor tendon tenosynovitis was evaluated palmarly. Tenosynovitis was scored using a binary system, with presence scored as 1 and absence as 0 [21,22]. Subclinical synovitis was defined as the presence of a GSUS synovitis score greater than 0 in a joint that was not clinically swollen on physical examination. The MSUS examination, including documentation, also required approximately fifteen minutes.

Following completion of the US assessment, several composite scores were calculated. These included the GS synovitis score (0–54 points), the PD synovitis score (0–54 points), and the total synovitis score, defined as the sum of the GS and PD synovitis scores (0–108 points). Tenosynovitis was summarized as a total score ranging from 0 to 10 points, representing the combined findings from the fourth extensor compartment and the flexor tendons. Salivary gland involvement was quantified using a GS score (0–12 points), a PD score (0–12 points), and a total salivary gland score representing their sum (0–24 points).

### 2.3. Statistical Methods

Data were analyzed using the statistical software program IBM SPSS Statistics for Windows (Version 28.0; Armonk, NY, USA: IBM Corp.). Continuous variables were assessed for normality with the Shapiro–Wilk test. For normally distributed variables, the central tendency was described with the mean and standard deviation (SD). Non-normally distributed variables were reported as median and interquartile range (IQR), and for these variables, between-group comparisons were performed through the Mann–Whitney U test. Associations between target variables were examined using Spearman’s rank-order correlation. The 95% confidence intervals for Spearman’s rho were calculated using the Fisher z-transformation. Since multiple Mann–Whitney U comparisons and Spearman rho correlations were performed, the significance level of 0.05 was adjusted using the Bonferroni correction to control for Type I error inflation. The adjusted alpha level is indicated in the notes beneath each table. Categorical and ordinal variables were presented as counts and percentages (%). All statistical tests were two-tailed with a 5% acceptable Type I error rate. Statistical significance was accepted at *p* < 0.05.

A post hoc power analysis using Monte Carlo simulation (10,000 iterations, Python Software Foundation. 2023. Python (Version 3.11.6) was applied to assess whether the observed between-group differences were detectable at the sample size of 60 patients and 20 healthy controls. A two-sided Mann–Whitney U test (α = 0.05) was applied at each iteration, and power was calculated as the proportion of iterations yielding *p* < 0.05. Effect size was expressed as the rank-biserial correlation (r). The obtained effect sizes for the primary outcome variables ranged from 0.75 to 0.99, with an estimated power exceeding 99%.

## 3. Results

### 3.1. Background Characteristics

This cross-sectional, multicenter study was conducted at the University Hospital “Kaspela” and the University Hospital “Pulmed” and included 60 patients with Sjögren’s syndrome and 20 healthy controls (HC). Women comprised 95% of the patient group and 100% of the control group. The two groups had similar median ages: 51 years (IQR 8) in patients versus 53 years (IQR 7) in HC (*p* = 0.325). The majority of patients (n = 37, 61.7%) were treated with hydroxychloroquine (HCQ): 20 as monotherapy and 17 in combination with other medications. Other therapies, used either alone or in combination, included methotrexate (MTX) in 20 (33.3%) patients (11 with HCQ and 9 as monotherapy), tocilizumab (TCZ) in 7 (11.7%) patients, azathioprine (AZA) in 6 (10.0%), rituximab (RTX) in 5 (8.3%), and corticosteroids (CS) in 4 (6.6%). A detailed breakdown of monotherapy and combination regimens, as well as corticosteroid dosage ranges, is provided in Supplementary Table S1. Clinical data about the patients are presented in Table 1.

The ESSDAI domain analysis showed that the most frequently affected domains were the articular (75%) and biological (50%) domains, followed by the glandular, lymphadenopathy, hematological, cutaneous, peripheral nervous system (PNS), and respiratory domains. The remaining domains were affected in fewer than 5% of patients, and no patient had central nervous system involvement (Table 2).

Inter-reader reliability between the two sonographers (TS and PT) was substantial to almost perfect for both GS and PD scores, with weighted κ values ranging from 0.60 to 0.80 across glands. Intra-reader reliability was similarly high, with weighted κ values between 0.70 and 0.85 for repeated readings. Agreement was slightly higher for GS scores than for PD scores, but all domains showed at least substantial reliability.

### 3.2. Ultrasound Assessment of Joints and Tendons in Patients and Healthy Controls

GSUS, PDUS, and total synovitis scores were significantly higher in the patient group than in the control group (all comparisons *p* < 0.001). The groups did not differ significantly in total tenosynovitis score at the adjusted alpha level of 0.0125 (obtained *p* = 0.041). Although the median value was 0 in both groups, individual scores in the patient group ranged from 0 to 2, whereas all scores in the control group were 0.

Of the 60 patients, 42 had no swollen joints. However, subclinical synovitis—defined as a GSUS synovitis score > 0—was detected in 66.7% (n = 28) of these patients (Table 3).

### 3.3. Synovitis at the Individual-Joint Level

**Figure 2:**
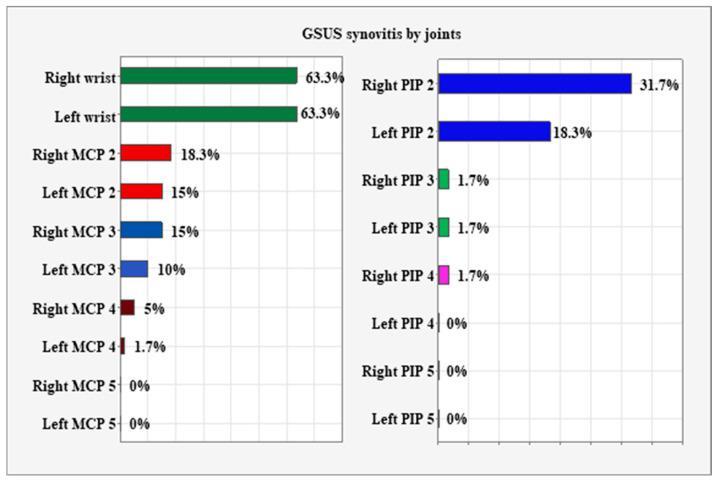
Distribution of GSUS synovitis at the individual-joint level. MCP—metacarpophalangeal, PIP—proximal interphalangeal.

GSUS synovitis assessed at the individual-joint level showed that the right and left wrists were the most frequently affected joints. This was followed by PIP2 (right and left) and then MCP2 and MCP3 (right and left)—Figure 2.

### 3.4. US Assessment of Salivary Glands in Patients and HC

GSUS, PDUS, and total salivary gland scores were significantly higher in the patient group compared with HCs (*p* < 0.001 for all comparisons) (Table 4). Most patients with pSS demonstrated grade 2 or 3 B-mode US abnormalities, indicating moderate to severe parenchymal involvement. All patients had at least one major salivary gland (MSG) with grade 2 or 3 changes, and every patient showed structural abnormalities in at least one submandibular gland. No patient exhibited isolated parotid gland involvement without concurrent submandibular gland abnormalities. In contrast, the reverse pattern was observed—parenchymal changes in the submandibular glands with a normal US appearance of the parotid glands.

### 3.5. Associations Between Joint and Salivary Glands US Scores

No significant relationships were found between the GSUS, PDUS, and total joint and salivary gland scores (Table 5).

### 3.6. Associations Between Sjögren’s Syndrome Disease Activity Indices and US Synovitis and Salivary Gland Scores

The ESSPRI total showed moderate positive associations with both the GSUS synovitis score (*p* = 0.002) and the total synovitis score (*p* = 0.003) (Table 6).

Table 7 presents the correlation coefficients between US synovitis scores and the three ESSPRI subcomponents (dryness, fatigue, and pain). The dominant factor in the relationships with US synovitis scores was the ESSPRI pain score, which showed strong positive correlations with GSUS and total synovitis scores (*p* < 0.001 for both) and a moderate positive correlation with PDUS synovitis (*p* < 0.001). The ESSPRI dryness score showed negative correlations with all three US synovitis scores; however, only the association with PDUS reached significance at the adjusted alpha level of 0.0167 (*p* = 0.008).

ESSDAI showed a low positive association with the GSUS salivary gland score, which was not significant at the adjusted alpha level of 0.0167 (obtained *p* = 0.030). No significant relationships were found between PDUS and the total salivary gland scores.

ESSPRI total showed significant positive associations with all US salivary scores (Table 8): GSUS salivary gland (*p* < 0.001), PDUS salivary gland score (*p* = 0.002), and total salivary gland score (*p* < 0.001).

### 3.7. US Salivary Gland Scores in Anti-Ro and IgG Positive and Negative Cases

The US salivary gland scores did not show significant associations with anti-Ro antibody positivity (Table 9) or hypergammaglobulinemia (Table 10).

## 4. Discussion

Recent advances in US technology, together with the growing expertise of rheumatologists in ultrasonography, have contributed to the expanding body of evidence supporting the use of US in the diagnosis, differential diagnosis, assessment of disease activity, and treatment monitoring of inflammatory, metabolic, and degenerative joint diseases, as well as systemic connective tissue disorders [6,7]. Sonographic criteria are now incorporated into the diagnostic work-up of several rheumatic diseases, including gout, polymyalgia rheumatica, and giant cell arteritis [23,24,25].

In pSS, US has become the imaging modality of choice for evaluating the salivary glands, musculoskeletal inflammation, and interstitial lung disease [26]. It enables detection of structural parenchymal changes in the MSG, facilitates early identification of non-Hodgkin lymphoma, guides core-needle biopsy of focal lesions, and allows assessment of joint synovitis and tendon involvement [26]. The OMERACT scoring system for structural abnormalities of the SMG and PG has demonstrated moderate reliability for GS findings and high reliability for CD changes [14,27,28]. Finzel et al. further confirmed substantial intra- and inter-reader reliability of the OMERACT SGUS grading system in patients with SS [29].

In this study, we sought to determine whether focal salivary gland abnormalities detected by US in patients with pSS reflect systemic disease activity as measured by ESSDAI and ESSPRI. A further objective was to explore the potential relationship between glandular and musculoskeletal involvement by assessing whether structural changes in the parotid and submandibular glands are associated with the presence of joint synovitis and tenosynovitis.

In our cohort, the median ESSDAI and ESSPRI scores were both 6, indicating moderate systemic disease activity and a high symptom burden as perceived by patients. These findings align with data from several large registries, including the Spanish GEAS-SS Registry, the GRISS group, and the Big Data Sjögren Project Consortium [30,31,32]. Analysis of ESSDAI domains in our patient cohort showed that the articular and biological domains were most frequently affected, followed by the glandular domain—again mirroring patterns reported in other large cohort studies [30,31,32].

The articular domain is among the most commonly involved in pSS, affecting approximately 53% of patients and presenting with manifestations ranging from arthralgia to symmetric non-erosive polyarthritis [33,34,35,36]. Carrubi et al. reported an even higher prevalence of articular involvement, reaching 86% [37]. In our study, 75% of patients exhibited joint involvement, likely reflecting the longer mean disease duration in our cohort. Subclinical synovitis—defined as a GSUS synovitis score > 0 and a total synovitis score > 0—was present in 66.7% of patients in our study. Grade 1 synovitis on GSUS and grade 0 on PDUS may be observed in healthy individuals or in the context of degenerative joint changes, particularly given the age of our study population [38]. However, according to the literature, most of these mild abnormalities—such as effusion or minimal synovial hypertrophy—are predominantly found in the feet, especially in the first metatarsophalangeal joint. In contrast, our US assessment focused exclusively on the hands, where such findings are less commonly reported in healthy controls. If this were a study involving patients with rheumatoid arthritis, we would certainly have applied a more stringent definition of subclinical synovitis. However, in pSS, synovitis is typically mild, and power Doppler positivity is rare. Therefore, the definition we used is appropriate for capturing the characteristic low-grade inflammatory changes seen in this patient population. Applying a stricter definition—requiring higher-grade GSUS findings in combination with PD positivity—would have resulted in an extremely low frequency of subclinical synovitis, which would not reflect the typical joint involvement pattern in pSS. Moreover, the patients included in our study had joint tenderness, and even GSUS grade 1 synovitis is clinically relevant in this context, as it may influence treatment decisions. In contrast, GS grade 1 findings in asymptomatic healthy controls are not clinically meaningful.

In our study, at the individual-joint level, GSUS most frequently detected synovitis in the wrists, followed by the second PIP joints. No patient exhibited grade 3 synovitis on GSUS, and only a small number showed PD positivity, typically grade 1. Tenosynovitis of the flexor tendons was also observed in a minority of patients.

These findings differ from those reported by Guedes et al., who identified grade 3 synovitis in 9.3% of pSS patients and tenosynovitis in 36.1% [39]. Amezcua-Guerra et al. found that synovitis most commonly affects the MCP and wrist joints in a symmetrical pattern, occurring in 76% of patients on GSUS. Meanwhile, PD positivity was relatively rare (12%) [34]. Carrubi et al. reported GSUS-detected synovitis in 27% of patients and PD positivity in 7%, predominantly in the hands and wrists, with tenosynovitis present in 17% [36]. Iagnocco et al. observed wrist synovitis in 37.5% of patients [35]. In a 2019 study by Lei et al., subclinical US-detected synovitis was identified in 21.7% of pSS patients—a considerably lower prevalence than in our cohort, likely reflecting the longer disease duration of the patients included in our study [36].

After joint involvement, glandular involvement represents the second most frequently affected ESSDAI domain in pSS. SGUS has demonstrated high sensitivity and specificity for the diagnosis of pSS, shows strong correlation with histopathology, assists in the early detection of salivary gland lymphoma, guides core-needle biopsy of focal lesions, and is sensitive to treatment-related changes [9,10,11,12,13]. Oshima et al. reported that PD signals within the salivary gland parenchyma decreased following initiation of immunosuppressive therapy [40,41]. Despite these promising findings, the responsiveness of SGUS to therapeutic interventions remains unclear and warrants further clarification through larger, prospective studies.

Several investigations have evaluated the diagnostic utility of the OMERACT scoring system. Fana et al. demonstrated that a cut-off of ≥2 in at least one MSG provides high sensitivity for fulfilling the 2016 ACR/EULAR classification criteria for SS [15]. Similarly, Rebel et al. showed that incorporating SGUS using the OMERACT score increases the sensitivity of the 2016 ACR/EULAR criteria when applying a threshold of ≥2 in at least one gland and ≥5 across all four glands (range 0–12) [42]. Tabaa et al. further reported that SGUS has a high negative predictive value, suggesting that patients with OMERACT scores < 2 in all major salivary glands and negative anti-SSA antibodies can be reliably excluded from an SS diagnosis without requiring salivary gland biopsy [43].

In our cohort, most patients exhibited moderate-to-severe B-mode abnormalities, with grade 2 or 3 changes in at least one of the four MSG. These findings are consistent with those reported by Zabotti et al. in 2025 [44]. All patients with pSS demonstrated structural abnormalities in at least one SMG. Notably, no patient showed isolated PG involvement without concurrent SMG changes, whereas the reverse pattern—abnormal SMG with normal PG—was frequently observed. This distribution aligns with observations by Cortes et al. and Theander et al., who reported that US abnormalities are most commonly detected in the SMG [45,46]. Theander et al. also found that PG US has excellent specificity (100%) but low sensitivity (up to 30%) for diagnosing pSS. The absence of isolated parotid involvement in our study may reflect anatomical differences between the glands, the higher susceptibility of the SMG to inflammatory changes, and the contribution of age-related chronic sialadenitis.

A key aim of our study was to determine whether the severity of parenchymal abnormalities in the MSG—scored semi-quantitatively using the OMERACT GSUS and Hocevar PDUS systems—correlates with joint and tendon inflammation, also assessed semi-quantitatively on GSUS and PDUS. No significant associations were identified between GS or PD synovitis scores, total synovitis scores, and any of the salivary gland US scores (GS, PD, or total SG score). To our knowledge, no previous studies have examined the relationship between articular and glandular inflammation assessed by US, making our findings an initial contribution to this area of investigation.

The second aim of this study was to determine whether glandular activity in pSS, as assessed by US, is related to systemic disease activity, as measured by the two composite indices ESSDAI and ESSPRI. ESSDAI demonstrated only a weak positive association with the GSUS salivary gland score, and no significant relationships were observed with PDUS or the total salivary gland score. These findings are consistent with those of Lei et al., who reported no correlation between total GS and PD synovitis scores and ESSDAI [36]. In contrast, Guedes et al. found that the number of joints with synovitis and tendons with tenosynovitis correlated with ESSDAI [39].

In our study, ESSPRI showed a different pattern. ESSPRI correlated with both joint and glandular involvement on US. The total ESSPRI score demonstrated moderate positive associations with the GSUS synovitis score and the total synovitis score, as well as significant positive associations with all salivary gland US scores—GSUS, PDUS, and total salivary gland score (*p* < 0.001). Another strength of our study is the inclusion of separate analyses for the ESSPRI pain, dryness, and fatigue subscales. These analyses showed that the ESSPRI pain score had strong positive correlations with both GSUS and total synovitis scores (*p* < 0.001 for each), as well as a moderate positive correlation with PDUS synovitis (*p* < 0.001), suggesting that pain in Sjögren’s syndrome is closely linked to underlying synovial inflammation. Targeting the key cytokines involved in synovitis is therefore likely to reduce the pain-related burden.

The discrepancy between ESSPRI and ESSDAI in their associations with US findings may be explained by the distinct dimensions of disease they capture. ESSPRI reflects symptoms directly experienced by patients and can be influenced by local glandular inflammation or joint discomfort, even in the absence of high systemic activity. Symptom–inflammation discordance is well recognized in Sjögren’s syndrome, and many patients report substantial symptom burden despite low systemic activity. SGUS detects local, organ-specific pathology, and ESSPRI aligns more closely with these localized changes.

ESSDAI, on the other hand, measures systemic activity and does not incorporate symptom burden. Its threshold-based scoring requires a certain degree of severity before domain scores increase, making it less sensitive to subtle musculoskeletal or glandular abnormalities. The musculoskeletal domain, for example, only scores synovitis when clinically evident. Mild or moderate synovitis may be subclinical—detectable by US but not by physical examination—and therefore not captured by ESSDAI. Moreover, ESSDAI excludes subjective symptoms such as fatigue, dryness, and pain, which are the primary drivers of ESSPRI. Local glandular or synovial inflammation may influence symptoms without being severe enough to elevate ESSDAI scores. Structural glandular changes detected on GSUS may also persist as damage even when systemic disease is inactive, further weakening the association with ESSDAI.

Previous studies have reported mixed findings. Schmidt et al. found no correlation between OMERACT-graded salivary gland changes and either ESSDAI or ESSPRI [47]. A Spanish study published in 2023 similarly reported no association between B-mode parenchymal abnormalities and ESSDAI [48]. In contrast, Inanc et al. observed that patients with systemic involvement had higher SGUS scores (Hocevar system) and more frequent severe structural changes (score ≥ 2) in both PG and SMG; these patients also had higher ESSPRI dryness scores [49]. Oshima et al. reported a correlation between PD activity in the salivary glands and ESSDAI [41]. Meanwhile, Zabotti et al. found an association between color Doppler scoring and ESSDAI, likely because they focused specifically on the glandular domain [44].

In our cohort, salivary gland US scores did not correlate with anti-Ro antibodies or hypergammaglobulinemia. This contrasts with findings by Schmidt et al., who reported associations between US-detected parenchymal abnormalities and both anti-Ro positivity and hypergammaglobulinemia [47].

Our study is the first to provide a comprehensive description of local glandular structural changes and joint and tendon involvement in pSS using high-frequency US. It is also the first to examine the relationship between glandular abnormalities and joint inflammation, both assessed semi-quantitatively with GSUS and PDUS. By including only patients with systemic disease activity, the study offers insight into how overall disease activity (ESSDAI) relates to localized glandular and musculoskeletal pathology.

Our findings have several implications for clinical practice. First, the lack of correlation between major salivary gland US abnormalities and systemic disease activity suggests that glandular structural damage progresses somewhat independently from systemic inflammation. Therefore, relying solely on systemic disease activity scores may underestimate the extent of glandular involvement. Second, the observed association between patient-reported symptom burden and both joint inflammation and salivary gland changes underscores the importance of incorporating patient-reported outcomes into routine evaluation. These measures may help clinicians identify patients with clinically meaningful inflammatory activity even when objective systemic disease activity indices appear low. Finally, the results support the use of targeted US assessment—both of joints and salivary glands—as a complementary tool to refine clinical judgment and guide individualized management strategies in pSS.

The absence of correlation between glandular and articular US findings suggests that these manifestations may reflect distinct underlying disease processes in pSS. One possible explanation is that the immunopathological mechanisms driving salivary gland destruction differ from those responsible for synovial inflammation. Glandular abnormalities detected on GSUS may largely represent chronic, cumulative structural damage rather than ongoing inflammatory activity, which would explain their weak association with systemic disease activity and ESSDAI scores. In contrast, synovitis is a more fluctuant feature that reflects current inflammatory activity and may vary over shorter time intervals. These differences in pathophysiology and temporal dynamics could account for the poor correlation observed between glandular and articular involvement. Future studies incorporating longitudinal imaging and histopathological data may help clarify the relationship between these two domains.

Several limitations warrant consideration. First, the relatively small sample size precluded stratification by treatment status, and the inclusion of participants receiving biologic therapy may have influenced the imaging outcomes. Although the sample size is modest, it is comparable to previous research in the field. In addition, a post hoc power analysis was performed to support the robustness of our findings.

Second, the cross-sectional design does not allow inferences regarding causality, highlighting the need for longitudinal investigations. Third, the absence of a comparative imaging modality, such as MRI, limits the ability to validate ultrasonographic findings against an established reference standard. In addition, the requirement for systemic manifestations beyond sicca symptoms, fatigue, and pain introduces a selection bias toward individuals with more pronounced systemic involvement, thereby limiting the external validity and generalizability of the results.

Although labial salivary gland biopsy is considered a diagnostic gold standard in pSS, only 24 patients in our cohort had undergone a biopsy, and these procedures were performed at varying time points prior to study inclusion. Because of the limited number of available biopsies and the heterogeneity in timing relative to the US and clinical assessments, we considered that a correlation analysis between biopsy status, disease activity measures, patient-reported outcomes, and US scores would not yield reliable or interpretable results. For this reason, biopsy findings were reported descriptively but were not incorporated into the correlation analyses. Future studies with larger, prospectively biopsied cohorts may help clarify the relationship between histopathological changes, patient-reported symptom burden, and imaging findings.

The inclusion criterion requiring systemic symptoms beyond sicca symptoms, fatigue, and pain introduces a degree of selection bias, as it favors recruiting patients with at least some systemic involvement. Consequently, our findings may not be fully applicable to pSS patients with predominantly glandular manifestations or those with very mild systemic symptoms. This limitation should be considered when interpreting the associations observed in this study. Future research, including the full spectrum of disease severity—ranging from purely glandular to highly systemic phenotypes—will be essential to determine whether these findings are generalizable across the broader pSS population.

Furthermore, the inclusion of patients receiving immunomodulatory therapies, including biologic agents, represents an important potential confounder. Treatments such as rituximab and tocilizumab are known to suppress synovitis and reduce power Doppler activity, potentially attenuating the true extent of musculoskeletal inflammation detectable by US. Consequently, the lack of correlation between glandular and articular US findings may in part reflect treatment-related suppression of joint inflammation rather than an absence of underlying disease activity. Although a sensitivity analysis excluding patients on biologic therapy would help clarify this issue, the number of biologic-treated patients in our cohort was too small to permit meaningful subgroup analyses. This limitation should be considered when interpreting the results, and future studies with larger, treatment-stratified cohorts will be important to validate these findings.

In addition, large-scale longitudinal studies involving more homogeneous patient populations would be better suited to elucidating the temporal dynamics of glandular alterations in relation to systemic disease activity.

Despite these limitations, the integration of SGUS and musculoskeletal US into the clinical management of pSS has the potential to support more personalized, patient-centered treatment strategies aimed at improving outcomes.

## 5. Conclusions

The severity of parenchymal abnormalities in the MSG, as assessed by US, does not correlate with articular inflammation or systemic disease activity in pSS. In contrast, patient-reported symptom burden shows significant associations with both joint inflammation and US-detected glandular changes. These findings highlight the importance of integrating patient-centered measures with imaging assessments. Future large-scale studies are required to confirm the clinical value of incorporating salivary gland ultrasonography into the routine evaluation of disease activity in pSS.

## Figures and Tables

**Table 1:** Patients’ demographic and clinical characteristics.

Variables		Statistics
Age o Median (IQR) o Minimum–Maximum		51 (8) 25–78
Sex n (%) o Men o Women		3 (5.0%) 57 (95.0%)
Symptom duration (years) o Median (IQR) o Minimum–Maximum		3 (2.0) 1–9
Tender joint count (TJC) o Median (IQR) o Minimum–Maximum		5 (5) 0–14
Swollen joint count (SJC) o Median (IQR) o Minimum–Maximum		0 (1) 0–8
Salivary gland biopsy n (%) o Yes o No		24 (40.0%) 36 (60.0%)
ANA IIF n (%) o 1:160 o 1:320 o 1:640 o 1:1280 o 1:2560		3 (5.0%) 9 (15.0%) 23 (38.3%) 23 (38.3%) 2 (3.4%)
Anti-Ro n (%) o Positive o Negative		50 (83.3%) 10 (16.7%)
Anti-La n (%) o Positive o Negative		19 (31.7%) 41 (68.3%)
Both anti-Ro and anti-La positive n (%)		19 (31.7%)
Both anti-Ro and anti-La negative n (%)		10 (16.7%)
Low C3 n (%) o Yes o No		10 (16.7%) 50 (83.3%)
Low C4 n (%) o Yes o No		31 (51.7%) 29 (48.3%)
High IgG (Hypergammaglobulinemia) n (%) o Yes o No		30 (50.0%) 30 (50.0%)
ESSDAI o Median (IQR) o Minimum–Maximum o Low (<5) o Medium (5–13) o High (≥14)		6 (1) 0–31 9 (15.0) 49 (81.7%) 2 (3.3%)
ESSPRI o Mean (SD) o Minimum–Maximum		6.29 (0.89) 4.30–8.60
ESSPRI Dryness (0–10) o Median (IQR) o Minimum–Maximum		6 (2) 4–9
ESSPRI Fatigue (0–10) o Median (IQR) o Minimum–Maximum		6 (1) 4–9
ESSPRI Pain (0–10) o Median (IQR) o Minimum–Maximum		6 (2) 3–9
Treatment	n (%)	
Hydroxychloroquine (HCQ):	37 (61.7%)	
Methotrexate (MTX):	20 (33.3%)	
Tocilizumab (TCZ):	7 (11.7%)	
Azathioprine(AZA):	6 (10.0%)	
Rituximab (RTX):	5 (8.3%)	
Corticosteroids (CS):	4 (6.6%)	

ESSDAI-EULAR Sjögren’s Syndrome Disease Activity Index; ESSPRI-EULAR Sjögren’s Syndrome Patient Reported Index.

**Table 2:** ESSDAI by domains.

ESSDAI by Domains	n (%) Affected Patients	Mean Score (SD)	Min.–Max.
Cutaneous	7 (11.7%)	0.40 (1.16)	0–6
Respiratory	5 (8.3%)	0.42 (1.39)	0–5
Renal	1 (1.7%)	0.08 (0.64)	0–5
Articular	45 (75.0%)	2.10 (1.53)	0–6
Muscular	2 (3.4%)	0.12 (0.78)	0–6
PNS	7 (11.7%)	0.67 (1.94)	0–10
CNS	0 (0.0%)	0.0 (0.0)	0–0
Glandular	11 (18.3%)	0.43 (0.98)	0–4
Constitutional	4 (6.7%)	0.20 (0.75)	0–4
Lymphadenopathy	11 (18.3%)	1.07 (2.19)	0–12
Hematological	8 (13.4%)	0.25 (0.65)	0–12
Biological	30 (50.0%)	0.50 (0.50)	0–1

PNS—peripheral nervous system; CNS—central nervous system.

**Table 3:** Joint and tendon US scores in patients and HC.

Variables	Patients (n = 60)	HC (n = 20)	Mann–Whitney U *p*-Value
GSUS synovitis o Median (IQR) o Minimum–Maximum	3 (4.0) 0–12	0.5 (1.0) 0–2	<0.001
PDUS synovitis o Median (IQR) o Minimum–Maximum	0 (0.0) 0–3	0 (0.0) 0–0	<0.001
Total synovitis score o Median (IQR) o Minimum–Maximum	3 (4.0) 0–14	0.5 (1.0) 0–2	<0.001
Total tenosynovitis score o Median (IQR) o Minimum–Maximum	0 (0.0) 0–2	0 (0.0) 0–0	0.041

Adjusted alpha level = 0.0125 (Bonferroni correction for three comparisons).

**Table 4:** Salivary gland scores in patients and HC.

US Salivary Gland Scores	Patients	HC	Mann–Whitney U *p*-Value
GSUS salivary gland score o Median (IQR) o Minimum–Maximum	7 (3.0) 4–12	1 (1.0) 0–2	<0.001
PDUS salivary gland score o Median (IQR) o Minimum–Maximum	3 (5.0) 0–12	0 (0.0) 0–0	<0.001
Total salivary gland score o Median (IQR) o Minimum–Maximum	10 (8.0) 4–24	1 (1.0) 0–2	<0.001

Adjusted alpha level = 0.0167 (Bonferroni correction for the three comparisons).

**Table 5:** Correlation results for joint and salivary gland GSUS and PDUS scores.

Variable 1	Variable 2	Spearman Rho Coefficient	95% CI	*p*-Value
GSUS synovitis	GSUS salivary gland	−0.061	−0.310–0.197	0.645
PDUS synovitis	PDUS salivary gland	−0.023	−0.278–0.234	0.862
Total synovitis score	Total salivary gland	0.110	−0.149–0.355	0.402

Adjusted alpha level = 0.0167 (Bonferroni correction for the three correlations).

**Table 6:** Correlation results for US synovitis scores and Sjögren’s syndrome disease activity indices.

Variable 1	Variable 2	Spearman Rho Coefficient	95% CI	*p*-Value
GSUS synovitis	ESSDAI	−0.120	−0.364–0.139	0.361
PDUS synovitis	ESSDAI	−0.091	−0.339–0.170	0.495
Total synovitis score	ESSDAI	−0.119	−0.363–0.140	0.365
GSUS synovitis	ESSPRI total	0.387	0.138–0.590	0.002
PDUS synovitis	ESSPRI total	0.141	−0.120–0.385	0.286
Total synovitis score	ESSPRI total	0.381	0.132–0.585	0.003

Adjusted alpha level = 0.0167 (Bonferroni correction for three correlations involving ESSDAI and ESSPRI total).

**Table 7:** Correlation coefficients between US synovitis scores and the three ESSPRI subcomponents (dryness, fatigue, and pain).

Variable 1	Variable 2	Spearman Rho Coefficient	95% CI	*p*-Value
GSUS synovitis	ESSPRI dryness	−0.275	(−0.498 to −0.018)	0.033
PDUS synovitis	ESSPRI dryness	−0.340	(−0.554 to −0.085)	0.008
Total synovitis score	ESSPRI dryness	−0.278	(−0.500 to −0.021)	0.032
GSUS synovitis	ESSPRI fatigue	0.133	(−0.127 to 0.375)	0.313
PDUS synovitis	ESSPRI fatigue	0.095	(−0.166 to 0.343)	0.474
Total synovitis score	ESSPRI fatigue	0.131	(−0.128 to 0.374)	0.317
GSUS synovitis	ESSPRI pain	0.811	(0.680 to 0.891)	<0.001
PDUS synovitis	ESSPRI pain	0.473	(0.234 to 0.659)	<0.001
Total synovitis score	ESSPRI pain	0.800	(0.665 to 0.885)	<0.001

Adjusted alpha level = 0.0167 (Bonferroni correction for three correlations within each subcomponent of ESSPRI).

**Table 8:** Correlation results for US salivary gland and Sjögren’s syndrome disease activity indices.

Variable 1	Variable 2	Spearman Rho Coefficient	95% CI	*p*-Value
GSUS salivary gland	ESSDAI	0.280	0.024 to 0.503	0.030
PDUS salivary gland	ESSDAI	0.236	−0.023 to 0.465	0.065
Total salivary gland	ESSDAI	0.248	−0.010 to 0.465	0.056
GSUS salivary gland	ESSPRI total	0.471	0.234 to 0.656	<0.001
PDUS salivary gland	ESSPRI total	0.391	0.143 to 0.593	0.002
Total salivary gland	ESSPRI total	0.438	0.196 to 0.630	<0.001

Adjusted alpha level = 0.0167 (Bonferroni correction for three correlations involving ESSDAI and ESSPRI total).

**Table 9:** US salivary gland scores for Anti-Ro positive and negative cases.

US Salivary Gland Scores	Anti-Ro	Median (IQR)	Min–Max.	Mann–Whitney U *p*-Value
GSUS salivary gland	Positive	7 (3)	5–12	0.157
	Negative	6 (5)	4–11	
PDUS salivary gland	Positive	3 (5)	0–12	0.561
	Negative	3 (5.25)	0–7	
Total salivary gland	Positive	10.5 (8)	5–24	0.325
	Negative	9 (9.5)	4–18	

Adjusted alpha level = 0.0167 (Bonferroni correction for three comparisons).

**Table 10:** US salivary gland scores for patients with hypergammaglobulinemia.

US Salivary Gland Scores	High IgG	Median (IQR)	Min–Max.	Mann–Whitney U *p*-Value
GSUS salivary gland	Yes	7 (3.25)	4–12	0.535
	No	7 (3.25)	5–12	
PDUS salivary gland	Yes	3 (5.25)	0–12	0.714
	No	3 (4.25)	0–9	
Total salivary gland	Yes	9.5 (8.5)	4–24	0.640
	No	10 (6.75)	5–21	

Adjusted alpha level = 0.0167 (Bonferroni correction for three comparisons).

## Data Availability

Raw data were generated at the University Hospital “Pulmed” and “Kaspela”. Derived data supporting the findings of this study are available from the corresponding author (T.S.) upon request.

## Supplementary Materials

The following supporting information can be downloaded at https://www.mdpi.com/article/10.3390/biomedicines14040819/s1. Table S1. Summary of Treatment Categories and Medication Use.
