# Supplementary material for: Ultrasound-Detected Salivary Gland and Joint Inflammation Strongly Reflect Patient-Perceived Symptom Burden in Primary Sjögren’s Syndrome: A Cross-Sectional Multicenter Study
Source: Biomedicines. 2026 Apr 3;14(4):819. doi: 10.3390/biomedicines14040819 (PMC13113349; doi:10.3390/biomedicines14040819)
Supplement: Supplementary file 1 [file biomedicines-14-00819-s001.zip › biomedicines-4209118-supplementary.pdf]

**Table S1. Summary of Treatment Categories and Medication Use**

| Treatment Category | Medication | n (%) | Monotherapy | Combination Therapy | Dosage Range |
|---|---|---|---|---|---|
| **Conventional synthetic DMARDs (csDMARDs)** | Hydroxychloroquine (HCQ) | 37 (61.7%) | 20 | 17 | — |
| | Methotrexate (MTX) | 20 (33.3%) | 9 | 11 (with HCQ) | — |
| | Azathioprine (AZA) | 6 (10.0%) | 4 | 2 | — |
| **Biologic therapies** | Tocilizumab (TCZ) | 7 (11.7%) | 5 | 2 | — |
| | Rituximab (RTX) | 5 (8.3%) | 5 | 0 | — |
| **Corticosteroids** | Prednisone/equivalent | 4 (6.6%) | 4 | 0 | 5–10 mg/day |
| **Other combinations** | HCQ + MTX | 11 (18.3%) | — | 11 | — |
| | HCQ + AZA | 2 (3.3%) | — | 2 | — |
| | HCQ + TCZ | 2 (3.3%) | — | 2 | |
